# Antibiotic-Resistant *Salmonella* Circulation in the Human Population in Campania Region (2010–2023)

**DOI:** 10.3390/antibiotics14020189

**Published:** 2025-02-12

**Authors:** Maria Francesca Peruzy, Nicoletta Murru, Maria Rosaria Carullo, Immacolata La Tela, Antonio Rippa, Anna Balestrieri, Yolande Thérèse Rose Proroga

**Affiliations:** 1 Department of Veterinary Medicine and Animal Production, University of Naples “Federico II”, Via Delpino 1, 80137 Naples, Italy; mariafrancesca.peruzy@unina.it (M.F.P.); murru@unina.it (N.M.); antonio_rippa@libero.it (A.R.); 2 Task Force on Microbiome Studies, University of Naples Federico II, 80138, Naples, Italy; 3 Department of Food Microbiology, Istituto Zooprofilattico Sperimentale del Mezzogiorno, Via Salute 2, 80055 Portici, Italy; mariarosaria.carullo@izsmportici.it (M.R.C.); immacolata.latela@izsmportici.it (I.L.T.); yolande.proroga@izsmportici.it (Y.T.R.P.)

**Keywords:** *Salmonella* spp., human isolates, antibiotic resistance, ciprofloxacin resistance, azithromycin resistance

## Abstract

**Background/Objectives**: A retrospective study was conducted to evaluate antibiotic resistance among *Salmonella* strains isolated during human infection using data from the computer database (SIGLA) of the *Salmonella* Typing Center (Ce.Ti.Sa) of the Istituto Zooprofilattico del Mezzogiorno (IZSM). **Methods**: From 2010 to 2023, the Ce.Ti.Sa laboratory tested 680 *Salmonella* strains against the following: amoxicillin/clavulanic acid, ampicillin, azithromycin, cefixime, cefoxitin, cefotaxime, ceftazidime, chloramphenicol, ciprofloxacin, colistin, erythromycin, gentamicin, kanamycin, meropenem, nalidixic acid, pefloxacin, streptomycin, sulfisoxazole, sulfonamides, tetracyclines, tigecycline, and trimethoprim. **Results**: The most common serovars were *S. monophasic Typhimurium* (23.2%), *S. Enteritidis* (16.8%), and *S. Typhimurium* (16.0%). Nearly all strains were resistant to azithromycin (99.4%) and showed high resistance to sulphonamides, tetracycline, streptomycin, and ampicillin. The study found that 45.8% of strains exhibited multidrug resistance. Resistance to ciprofloxacin increased over time. Serovar-specific resistance varied: *S. monophasic Typhimurium* was resistant to azithromycin (100.0%), tetracycline (93.0%), and ampicillin (92.4%); *S. Enteritidis* showed 100.0% resistance to azithromycin; *S. Typhimurium* had high resistance to azithromycin, streptomycin, and ampicillin; and *S. Infantis* was resistant to erythromycin, sulfonamides, and azithromycin. **Conclusions**: The study highlights a troubling prevalence of *Salmonella*-resistant strains, emphasizing the need for infection prevention, proper antibiotic use in humans and animals, and the development of new antibiotics.

## 1. Introduction

*Salmonella* is a genus of Gram-negative rod-shaped bacteria that includes two species: *S. enterica* and *S. bongori*. *S.enterica* is subdivided into six sub-species: *S.enterica* subsp. *enterica*, *S. enterica* subsp. *salamae*, *S.enterica* subsp. *arizonae*, *S.enterica* subsp. *diarizonae*, *S. enterica* subsp. *indica*, and *S. enterica* subsp. *houtenae. S. enterica* subsp. *enterica* is the most commonly isolated sub-species from humans during infections [1].

*Salmonella enterica* comprises more than 2600 serovars, which are subdivided into two groups based on their pathogenic behaviors: typhoidal and non-typhoidal. *Salmonella* spp., depending on the serotype and the host involved, can cause asymptomatic infections or result in illnesses with severe symptoms [2]. Infections caused by typhoid salmonellae are known for their high mortality rates in humans, while infections caused by non-typhoidal salmonellae usually cause self-limiting gastroenteritis [3]. The groups most at risk of contracting a *Salmonella* infection include children under 5 years old and adults aged 65 and older [4].

Humans can be infected by *Salmonella* spp. through contact with infected animals or with contaminated food or water [2]. In the European Union (EU), salmonellosis is the second most common zoonosis after campylobacteriosis, with 65,208 human cases confirmed in 2022. The top five serovars involved in these infections were as follows: *S. Enteritidis* (67.3%), *S. Typhimurium* (13.1%), *S. Typhimurium monophasic* (4.3%), *S. Infantis* (2.3%), and *S. Derby* (0.9%) [5].

The food categories most involved in salmonellosis outbreaks in humans were eggs and egg products (86 outbreaks), mixed food (24 outbreaks), pig meat and products thereof (18 outbreaks), sweets and chocolates (12 outbreaks), and bakery products (11 outbreaks) [5].

Salmonellosis in humans, if caused by non-typhoidal strains, usually has a benign course with spontaneous recovery. However, it can be fatal among vulnerable categories of people such as children, the elderly, pregnant women, and the immunocompromised. In this case, antibiotic therapy with fluoroquinolones and third-generation cephalosporins is required [3,6].

According to the World Health Organization, antimicrobial resistance (AMR) is one of the most important public health threats of the 21st century. It is predicted that this threat could affect millions of people worldwide in the future [3]. AMR infections are more likely to lead to bloodstream infections and higher hospitalization rates, likely due to their invasive nature and the limited effectiveness of standard treatments [7]. High levels of resistance among *Salmonella* strains isolated from humans have been reported worldwide [8,9,10], including in Europe.

In Germany, for example, strains of *Salmonella* have been recorded as sensitive or intermediately resistant to tetracyclines, aminoglycosides, most beta-lactam antibiotics, quinolones, co-trimoxazole group antibiotics, chloramphenicol, nitrofurantoin, and azithromycin [11].

Although Italy has implemented several strategic measures to manage resistant infections effectively and optimize antibiotic usage, such as the National Action Plan on Antimicrobial Resistance (PNCAR) and clinical guidelines, it remains one of the most AMR-affected countries in Europe. Alarmingly high levels of AMR have been reported in hospitals and across various regions of the country [12].

This concerning status highlights the urgent need for targeted interventions to address AMR, which poses significant challenges to public health by reducing the efficacy of treatments for bacterial infections [12]. Recent data on the antimicrobial susceptibility of *Salmonella* isolates from humans in the Campania region are not available. While salmonellosis burden appears low in Campania, hospitalization rates exceed the EU average [4]. Therefore, studying antibiotic resistance in this region is essential.

The study aimed to evaluate the antibiotic resistance among *Salmonella* strains isolated during human infection using data from the computer database (SIGLA) of the *Salmonella* Typing Center (Ce.Ti.Sa) of the Istituto Zooprofilattico del Mezzogiorno (IZSM) from 2010 to 2023.

## 2. Results

### 2.1. General Findings

Between 2010 and 2023, 680 *Salmonella* strains were analyzed, of which 52.6% were isolated from males and 41.2% from females (Supplementary Material S1). No information regarding the sex was recorded for 42 isolates. The age of patients ranged from 0 to 85 years, but demographic data were missing for 386 cases. Strains were primarily obtained from fecal samples (90.0%), with smaller proportions from blood (6.8%) and urine (1.2%). No information regarding the sample of origin was recorded for 14 isolates. Hospitalization was reported for 78.4% of cases (Supplementary Material S1).

### 2.2. Serovar Distribution

Strains belonged to 63 serovars, with *S. monophasic Typhimurium* (*n* = 158, 23.2%), *S. Enteritidis* (*n* = 114, 16.8%), *S. Typhimurium* (*n* = 109, 16.0%), *S. Napoli* (*n* = 39, 5.7%), and *S. Infantis* (*n* = 37, 5.4%) being the most prevalent (Table 1). No information regarding the serovar was recorded for 12 isolates. The distribution of serovars did not significantly differ between males and females, except for *S. Derby*, which was more common in females (6.1%, *p* < 0.05), and *S. Brandenburg*, more common in males (2.8%, *p* < 0.05). Although information regarding the age of the patients for around half of the strains was not available, based on the available data, the prevalence of monophasic Typhimurium 1.4.[5].12:I:- (27.3%) and Typhimurium (26.5%) was higher in children than in adults (monophasic Typhimurium 1.4.[5].12:I:- : 14,.6% and Typhimurium: 13.1%) (*p* < 0.05).

The most prevalent serovar isolated from feces and blood was of monophasic Typhimurium 1.4.[5].12:I:- (feces: 23.9% and blood: 15.2%), whereas Typhimurium (25.0%) and Infantis (25.0%) were more frequently isolated from urine samples. However, it is important to note that the number of urine samples was relatively low (Supplementary Material S1).

### 2.3. Antibiotic Resistance

**Figure 1:**
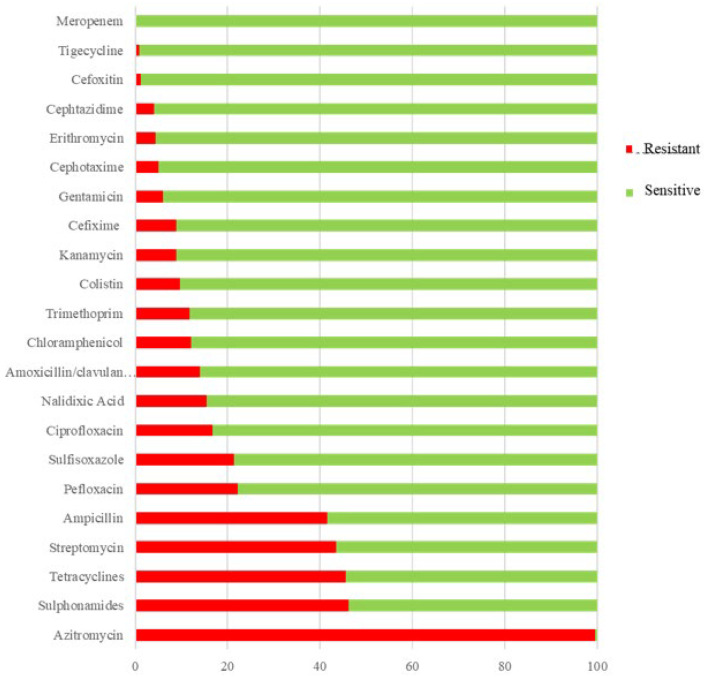
Percentages indicating the resistance and sensibility of the *Salmonella* strains.

Almost all strains were resistant to azithromycin (*n* = 339/341, 99.4%, 95% CI: 100.2, 98.6). High resistance was also observed for sulphonamides (*n* = 156/338, 46.2%, 95% CI: 51.5, 40.8), tetracycline (*n* = 310/681, 45.5%, 95% CI: 49.3, 41.8), streptomycin (*n* = 150/345, 43.5%, 95% CI: 48.7, 38.3), and ampicillin (*n* = 283/681, 41.6%, 95% CI: 45.3, 37.9). In contrast, all strains were sensitive to meropenem (Figure 1). Resistance to ciprofloxacin increased significantly over the study period (*p* < 0.05), while resistance to chloramphenicol and tetracyclines decreased (*p* < 0.05) (Supplementary Material S2).

A total of 99 strains were sensitive to all antibiotics, whilst a total of 45.8% (*n* = 318) of strains were classified as MDR, with some exhibiting resistance to up to 16 antibiotics.

No significant differences in resistance patterns were observed among the different age groups (children, adults, and elderly people) (*p* > 0.05). No significant differences in resistance patterns were observed between strains isolated from feces and blood (*p* > 0.05), except for multidrug resistance patterns (MDR: feces, 47.9% vs. blood, 23.9%) (*p* < 0.05).

With serovar, during the period under investigation, not all strains were tested against all the antibiotics. *S. monophasic Typhimurium* showed high resistance to azithromycin (*n* = 95/95, 100.0%), followed by tetracycline (*n* = 146/157, 93.0%, 95% CI: 97.0, 89.0) and ampicillin (*n* = 145/157, 92.4%, 95% CI: 96.5, 88.2) (Table 2). Strains belonging to *S. enteritidis* were highly resistant to azithromycin (*n* = 57/57, 100.0) (Table 2).

Strains belonging to *S. Typhimurium* were highly resistant to azithromycin (*n* = 23/23, 100.0%), followed by streptomycin (*n* = 55/86, 63.9%,95% CI: 53.8, 74.1) and ampicillin (*n* = 69/109, 63.3%, 95% CI: 54.3, 72.4) (Table 2).

Strains belonging to *S. Napoli* were highly resistant to azithromycin (*n* = 13/13, 100.0%) (Table 2).

Strains belonging to *S. Infantis* were highly resistant to erythromycin (*n* = 5/5, 100.0%), sulfisoxazole (*n* = 4/4, 100.0%), sulphonamides (*n* = 10/12, 83.3%, 95% CI: 104.4, 62.3), and azithromycin (*n* = 15/21, 71.4%,95% CI: 90.6, 52.1) (Table 2).

Moreover, a high percentage of strains belonging to *S. Typhimurium* (*n* = 139, 88.0%), *S. Typhimurium* (*n* = 71, 65.1%), and *S. Infantis* (*n* = 20, 54.1%) were MDR.

Statistical differences in resistance between the different serovars are reported in Table 2. In general, the resistance profiles for the serovars were similar, except for *S. Infantis*, which showed the highest difference.

## 3. Discussion

*Salmonella* infection is the most prevalent foodborne illness in Italy, with the Campania region (southern Italy) being a key focus due to its high hospitalization rates [13]. This study evaluated the AMR patterns of *Salmonella* serotypes isolated from humans in this region, highlighting significant concerns regarding MDR and the public health implications of antibiotic misuse.

Antimicrobial resistance is primarily driven by the inappropriate and excessive use of antibiotics [14]. In Italy, antibiotic consumption has decreased from 20.3 defined daily doses (DDDs) per 1000 inhabitants per day in 2013 to 17.2 DDDs in 2023, reflecting gradual improvements in antimicrobial stewardship (see supporting data at https://www.statista.com/statistics/941940/consumption-of-antibiotics-in-italy/ accessed on 14 January 2025). However, the present study underscores persistent resistance issues, particularly to azithromycin, sulphonamides, tetracyclines, streptomycin, and ampicillin.

Azithromycin resistance was detected in almost all strains, posing a major concern as this antibiotic, alongside meropenem, represents a last-line treatment for MDR *Salmonella* infections [15]. The resistance mechanism is traced to a point mutation (R717Q/L) in the efflux pump encoded by the acrB gene, complicating infection management and treatment outcomes [16]. Fortunately, no resistance to meropenem was observed, providing some reassurance for its continued efficacy.

Resistance to sulphonamides, tetracyclines, streptomycin, and ampicillin was consistent with previous studies, such as Proroga et al.’s (2018), which reported high resistance rates of tetracycline (64.0%), streptomycin (62.0%), sulphonamide (57.3%), and ampicillin (56.0%) [17]. These findings are linked to the widespread use of these antibiotics in food-producing animals, resulting in environmental contamination through animal excretions, aquaculture, plant production, and waste streams from antibiotic manufacturing. This environmental exposure facilitates the persistence and transfer of antibiotic residues and resistance genes to pathogenic bacteria, intensifying the AMR crisis [18]. However, no information regarding the genomic resistance profile is available because, in this retrospective study, data were extracted from the IZSM computer database (SIGLA) of Ce.Ti.Sa. at IZSM. Over the years, Ce.Ti.Sa. has served regional hospitals solely by providing them with the phenotypic resistance profile.

The environmental contamination from wastewater can contribute to the accumulation of antibiotic residues, as these substances are not fully degraded during water treatment processes before being released into the environment. This contributes to the rise in antibiotic-resistant bacterial strains [19,20].

Improper antibiotic practices, including self-medication, incorrect dosages, and the premature discontinuation of treatment, further exacerbate resistance to tetracycline, ampicillin, and streptomycin [21,22].

Tetracycline resistance in *Salmonella* arises through mechanisms such as efflux pumps, rRNA target modification, and compound inactivation. Sulphonamide resistance stems from altered target sites (mutations in DHPS), enzyme overproduction, or bypass pathways. Similarly, aminoglycoside resistance involves enzymatic modifications, efflux pumps, or antibiotic degradation. These multifaceted resistance mechanisms underscore the adaptability of *Salmonella* in evading antibiotic action [23].

Nearly half of the *Salmonella* strains in this study exhibited MDR, posing a severe public health threat due to higher mortality rates and prolonged hospital stays associated with MDR infections [24,25]. Indeed, it has been demonstrated that multidrug-resistant *Salmonella* infections may pose a more serious impact on human health compared to infections caused by less resistant strains [26].

An increase in the resistance to ciprofloxacin was observed over the last year. This antibiotic is used for treating life-threatening infections, and the increasing percentage of resistance could be explained by the massive use of this antibiotic in clinical practice [27]. The rising resistance to ciprofloxacin, a first-line treatment for life-threatening infections, is particularly concerning and is associated with its widespread clinical use [3].

Interestingly, resistance to chloramphenicol has declined, likely due to its prohibition in food-producing animals and its association with severe side effects, such as aplastic anemia [14,28]. However, despite this prohibition, monitoring remains necessary, as antibiotic resistance can continue to increase even when specific antibiotics are no longer in use. This phenomenon can be explained by the fact that bacteria can develop cross-resistance to multiple antibiotics simultaneously [14].

Colistin resistance was moderate (9.7%), comparable to previous Italian data (7.7%). While colistin’s use in human medicine was previously limited due to toxicity, its reintroduction as a last-resort treatment for MDR infections raises concerns about the potential for increasing resistance [29].

With serovar, in the present study, strains belonging to *S. monophasic Typhimurium* showed high resistance against tetracycline and ampicillin. Results were in line with those reported by Qin et al. (2022) (tetracycline: 93.0%; ampicillin = 91.0%) [21].

Strains belonging to *S.* Typhimurium were highly resistant to azithromycin, followed by streptomycin and ampicillin. The resistance shown by *S. typhimurium* against azithromycin was in contrast to those reported by Chiou et al. (2023) (4.2%) [30]. However, the results aligned with those observed by Chiou et al. (2023) regarding streptomycin (73.7%), ampicillin (74.9%), and tetracyclines (82.2%) [30].

The strains belonging to *S. Enteritidis* were resistant against ciprofloxacin. This is in contrast to the study by Dai et al. (2021) [31], in which no resistance was registered.

Except for azithromycin, the serovar Napoli was moderately resistant against the other antibiotics. This serovar is frequently detected in Italy and is gaining increasing relevance as a human pathogen in Europe [17]. Therefore, it is important to conduct further research to estimate the antibiotic resistance rate of this emerging serovar.

The serovar Infantis is commonly isolated from poultry meat and is one of the top five serovars responsible for human infection in the EU [32]. The same study demonstrated that the increase in *Salmonella* Infantis strains resistant to sulfonamides, tetracyclines, and ciprofloxacin may be linked to the spread of the pESI-like plasmid-harboring clone, which belongs to one of the two main *S. Infantis* populations circulating in Italy [32].

The prevalence of MDR strains, particularly in monophasic Typhimurium (68.2% MDR: *n* = 1668), aligns with national data indicating that 22.1% of human *Salmonella* isolates in 2022 were MDR [33]. This emphasizes the urgent need for robust surveillance and targeted interventions to mitigate AMR.

## 4. Materials and Methods

Data on resistant *Salmonella* strains and the epidemiological information of patients were extracted from the IZSM computer database (SIGLA) of the Ce.Ti.Sa. of the IZSM. SIGLA is a cloud-based laboratory information management system (LIMS) used for managing the entire laboratory process of the Italian Zooprophylactic Institutes, including automatic acceptance, sample analysis, entry of results, and the generation of test reports.

At Ce.Ti.Sa., human strains are collected from different hospitals in the Campania region and are serotyped according to the Kaufmann–White scheme [34]. Throughout these years, the antimicrobial susceptibility of the isolates was determined using the disk diffusion method, following the Clinical and Laboratory Standards Institute (CLSI) recommendations, and a quality control strain (Escherichia coli ATCC 25922) was always included in the test. The number of strains tested for each antibiotic is reported in Table 3.

In the present retrospective study, data were collected as per *Salmonella* serovars, antibiotics, year (2010–2023), age, sex, and biological specimens (Supplementary Material S1). Moreover, the number of people hospitalized was also recorded. In evaluating the results, strains displaying intermediate resistance were regarded as resistant, while those showing resistance to at least three antibiotic classes were considered multidrug-resistant (MDR) [35].

### Statistical Analysis

The collected data were recorded using Microsoft^®^ Excel^®^ 2018. Differences in drug and multidrug resistance among *Salmonella* serovars, antibiotics, years, age groups (children: 0–5 years, adults: 6–65 years, and elderly: 66+ years), sex, and biological specimens were analyzed using the chi-squared test (χ^2^) in Microsoft^®^ Excel^®^.

## 5. Conclusions

In conclusion, this study found a high proportion of *Salmonella* spp. strains resistant to antimicrobials. Resistance to sulphonamides, tetracyclines, and azithromycin has reached concerning levels. To address the concerning levels of antimicrobial resistance in *Salmonella* spp., several targeted interventions can be implemented. These include the establishment of antimicrobial stewardship programs (ASPs), the aims of which include strengthening infection prevention and control measures, optimizing diagnostic testing and surveillance, limiting the use of critically important antibiotics, and encouraging research and development. However, no information regarding the genomic resistance profile has been collected from these strains yet. Further research on the molecular profiles of these antibiotic-resistant bacteria (ARB) is needed to acquire a deeper understanding of their resistance mechanisms.

## Figures and Tables

**Table 1:** Frequency of *Salmonella* serovar isolation from human samples (2010–2023).

	Serovar	n.	%	CI (95%)			Serovar	n.	%	CI (95%)	
1	Monophasic Typhimurium	158	23.2	26.41	20.06	34	Szentes	2	0.3	0.70	−0.11
2	Enteritidis	114	16.8	19.57	13.96	35	Adjame	1	0.1	0.44	−0.14
3	Typhimurium	109	16.0	18.79	13.27	36	Agbeni	1	0.1	0.44	−0.14
4	Napoli	39	5.7	7.48	3.99	37	Anatum	1	0.1	0.44	−0.14
5	Infantis	37	5.4	7.15	3.74	38	Apeyeme	1	0.1	0.44	−0.14
6	Derby	29	4.3	5.78	2.75	39	Chingola	1	0.1	0.44	−0.14
7	Rissen	18	2.6	3.85	1.44	40	Corvallis	1	0.1	0.44	−0.14
8	Brandenburg	11	1.6	2.57	0.67	41	Duesseldorf	1	0.1	0.44	−0.14
9	Panama	10	1.5	2.38	0.57	42	Edinburg	1	0.1	0.44	−0.14
10	Typhi	10	1.5	2.38	0.57	43	Fischerhuette	1	0.1	0.44	−0.14
11	Give	9	1.3	2.18	0.46	44	Group4	1	0.1	0.44	−0.14
12	Bovismorbificans	8	1.2	1.99	0.37	45	Hvittingfoss	1	0.1	0.44	−0.14
13	London	8	1.2	1.99	0.37	46	Isangi	1	0.1	0.44	−0.14
14	Saintpaul	8	1.2	1.99	0.37	47	Kapemba	1	0.1	0.44	−0.14
15	Agona	7	1.0	1.79	0.27	48	Kingstom	1	0.1	0.44	−0.14
16	Coeln	6	0.9	1.59	0.18	49	Lagos	1	0.1	0.44	−0.14
17	Litchfield	6	0.9	1.59	0.18	50	Loanda	1	0.1	0.44	−0.14
18	Newport	5	0.7	1.38	0.09	51	Mbandaka	1	0.1	0.44	−0.14
19	Goldcoast	4	0.6	1.16	0.01	52	Meleagridis	1	0.1	0.44	−0.14
20	Sandiego	4	0.6	1.16	0.01	53	Meston	1	0.1	0.44	−0.14
21	Strathcona	4	0.6	1.16	0.01	54	Mishmarhaemek	1	0.1	0.44	−0.14
22	Thompson	4	0.6	1.16	0.01	55	Miyazaki	1	0.1	0.44	−0.14
23	Bredeney	3	0.4	0.94	−0.06	56	Montevideo	1	0.1	0.44	−0.14
24	Livingstone	3	0.4	0.94	−0.06	57	Nyborg	1	0.1	0.44	−0.14
25	Muenchen	3	0.4	0.94	−0.06	58	Oranienburg	1	0.1	0.44	−0.14
26	Pomona	3	0.4	0.94	−0.06	59	Paratyphi B	1	0.1	0.44	−0.14
27	Stanley	3	0.4	0.94	−0.06	60	Poona	1	0.1	0.44	−0.14
28	Subspii	3	0.4	0.94	−0.06	61	Singapore	1	0.1	0.44	−0.14
29	Kasenyi	2	0.3	0.70	−0.11	62	Urbana	1	0.1	0.44	−0.14
30	Kottbus	2	0.3	0.70	−0.11	63	Virchow	1	0.1	0.44	−0.14
31	Manhattan	2	0.3	0.70	−0.11	63	Worthington	1	0.1	0.44	−0.14
32	Muenster	2	0.3	0.70	−0.11		N.I.	12	1.8		
33	Stanleyville	2	0.3	0.70	−0.11						

N.I.: no information; CI: confidence interval.

**Table 2:** Antibiotic sensitivity and resistance of common Salmonella serovars.

	AMC-C	AMP	AZM	CFM	CFX	CTX	CAZ	CHL	CIP	CL	ERY	GEN	KAN	MPM	NAL	PFX	SPT	SFX	SPH	TET	TG	TMP
*S. monophasic Typhimurium*																						
R	11 ^a^	145 ^a^	95 ^a^	7 ^a^	0 ^a^	6 ^a^	5 ^a^	15 ^a^	16 ^a^	0 ^a^	1 ^a^	20 ^a^	7 ^a^	0 ^a^	16 ^a^	0 ^a^	53 ^a^	0 ^a^	54 ^a^	146 ^a^	0 ^a^	12 ^ac^
S	51	12	0	55	58	151	152	142	140	95	0	136	48	95	141	0	9	0	8	11	95	140
*S. Enteritidis*																						
R	0 ^b^	4 ^b^	57 ^a^	2 ^a^	0 ^a^	1 ^a^	1 ^a^	5 ^a^	19 ^a^	24 ^b^	2 ^a^	2 ^b^	0 ^a^	0 ^a^	18 ^c^	0 ^a^	1 ^b^	0 ^a^	3 ^b^	7 ^b^	0 ^a^	2 ^a^
S	54	103	0	48	55	107	107	102	88	32	9	106	50	53	90	0	53	0	47	101	56	93
*S. Typhimurium*																						
R	29 ^c^	69 ^c^	23 ^a^	7 ^a^	3 ^a^	5 ^a^	5 ^a^	26 ^b^	15 ^a^	0 ^a^	2 ^a^	3 ^b^	7 ^a^	0 ^a^	16 ^c^	0 ^a^	55 ^c^	0 ^a^	59 ^cd^	62 ^d^	1 ^a^	15 ^c^
S	58	40	0	79	75	104	104	83	94	23	36	106	79	23	93	0	31	2	27	47	22	84
*S. Napoli*																						
R	0 ^ab^	2 ^b^	13 ^a^	2 ^a^	0 ^a^	1 ^a^	1 ^a^	1 ^a^	4 ^a^	0 ^a^	0 ^a^	2 ^a^	0 ^a^	0 ^a^	1 ^a^	0 ^a^	0 ^b^	0 ^a^	1 ^b^	3 ^b^	0 ^a^	1 ^c^
S	27	37	0	24	27	38	38	38	35	13	7	37	26	13	38	1	26	0	25	36	13	33
*S. Infantis*																						
R	0 ^ab^	18 ^c^	15 ^b^	7 ^b^	0 ^a^	12 ^b^	9 ^b^	3 ^a^	15 ^b^	1 ^a^	5 ^a^	1 ^a^	6 ^b^	0 ^a^	18 ^b^	4 ^a^	5 ^d^	4 ^a^	10 ^ad^	20 ^d^	0 ^a^	12 ^b^
S	16	16	6	5	16	22	25	31	19	21	0	33	6	21	16	2	8	0	2	14	21	22

Values in columns with different lowercase letters indicate statistically significant differences (*p* < 0.05). Amoxicillin/clavulanic acid (AMC-C), ampicillin (AMP), azithromycin (AZM), cefixime (CFM), cefoxitin (CFX), cephotaxime (CTX), cephtazidime (CAZ), chloramphenicol (CHL), ciprofloxacin (CIP), colistin (CL), erythromycin (ERY), gentamicin (GEN), kanamycin (KAN), meropenem (MPM) nalidixic acid (NAL), pefloxacin (PFX), streptomycin (SPT), sulfisoxazole (SFX), sulphonamide (SPH), tetracycline (TET), tigecycline (TG), and trimethoprim (TMP).

**Table 3:** Number of strains tested for the different antibiotics.

Antibiotics	N. of Strains Tested
Ampicillin	678
Cephotaxime	680
Cephtazidime	679
Cefixime	336
Cefoxitin	350
Amoxicillin/clavulanic acid	360
Nalidixic acid	679
Gentamicin	677
Streptomycin	343
Kanamycin	335
Sulphonamides	336
Trimethoprim	624
Chloramphenicol	678
Tetracyclines	679
Ciprofloxacin	676
Tigecycline	338
Erythromycin	67
Colistin	339
Azithromycin	341
Meropenem	335
Pefloxacina	18
Sulfisoxazole	14

## Data Availability

The data presented in this study are available from the corresponding author upon reasonable request.

## Supplementary Materials

The following supporting information can be downloaded at: https://www.mdpi.com/article/10.3390/antibiotics14020189/s1, S1: Table S1. Foglio 1; S2: Table S2. Foglio 1.
